# Beyond the Benefits: A Case Study on the Complications of Sodium-Glucose Co-Transporter-2 (SGLT2) Inhibitors (Euglycemic Diabetic Ketoacidosis (DKA) and Takotsubo Cardiomyopathy)

**DOI:** 10.7759/cureus.55068

**Published:** 2024-02-27

**Authors:** Rand Sabanci, Moiz Saaed, Appa Bandi, Kathryn Das, Matthew Wilcox

**Affiliations:** 1 Internal Medicine, Michigan State University, East Lansing, USA; 2 Cardiology, Sparrow Hospital Thoracic and Cardiovascular Institute, Lansing, USA

**Keywords:** medication side effect, side effect, heart failure, canagliflozin, takotsubo cardiomyopathy (tc), euglycemic diabetic ketoacidosis, sglt2 inhibitors

## Abstract

Sodium-glucose co-transporter-2 (SGLT2) inhibitors, integral in type 2 diabetes mellitus (T2DM) management, are not without risks, with reported adverse effects including euglycemic diabetic ketoacidosis (EDKA). We present a case of a 75-year-old female with T2DM on canagliflozin, who developed altered mental status (AMS), nausea, vomiting, and hypotension. The laboratory results revealed ketoacidosis, elevated troponins, and Takotsubo cardiomyopathy (TC), prompting the cessation of canagliflozin. This paradoxical EDKA case underscores the necessity for cautious prescribing. Additionally, our discussion delves into the risk factors, mechanisms, and epidemiology of EDKA associated with SGLT2 inhibitors (SGLT2i), emphasizing the importance of individualized medicine and shared decision-making in their use, despite their proven cardiovascular benefits.

## Introduction

Sodium-glucose co-transporter-2 (SGLT2) inhibitors are designed to lower the plasma glucose level by inhibiting sodium (Na+)-glucose-coupled transport in the proximal renal tubule [1]. Multiple side effects have been reported including urogenital infections, dehydration, and euglycemic diabetic ketoacidosis (EDKA). Ketogenesis results from the metabolic transition from utilizing lipids instead of glucose as a result of the elevated glucagon-to-insulin ratio promoted by SGLT2 inhibitors (SGLT2i) [2]. SGLT2 inhibitors are currently a part of guideline-directed medical therapy (GDMT) with an evident reduction of cardiovascular death and hospitalizations for heart failure (HF) [3].

## Case presentation

A 75-year-old female patient with a past medical history of type 2 diabetes mellitus (T2DM) on canagliflozin, hypertension, and hyperlipidemia presented to the emergency department with altered mental status (AMS), nausea, vomiting, and decreased appetite. The patient was tachycardic with a heart rate of 114 beats per minute (bpm) and hypotensive with blood pressure (BP) of 95/68. Laboratory workup revealed elevated troponins at 335 ng/mL, brain natriuretic peptide (BNP) of 1,072 pg/mL, pH of 6.99, bicarbonate level of 5 mmol/L, anion gap of 41, blood glucose level at 246 mg/dL, and beta-hydroxybutyrate of 82 mmol/L, which are summarized in Table 1.

**Table 1:** Initial laboratory investigations BNP: brain natriuretic peptide

Investigation	Value	Normal range
Troponin	335 ng/mL	0-18 ng/mL
BNP	1,072 pg/mL	0-100 pg/mL
pH	6.99	7.35-7.45
Bicarbonate	5 mmol/L	20-32 mmol/L
Anion gap	41	2-16
Blood glucose	246 mg/dL	65-99 mg/dL
Beta-hydroxybutyrate	82 mg/dL	<4.1 mg/dL

**Figure 1:**
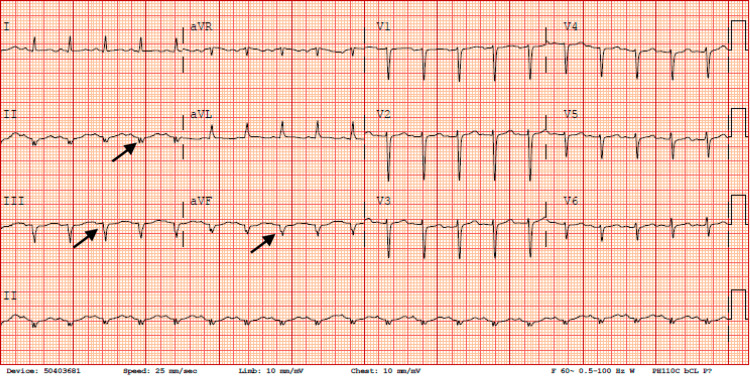
EKG showing sinus tachycardia with an old inferior infarct indicated by the arrows

An imaging of the head showed no acute process. EKG showed sinus tachycardia with an old inferior infarct (Figure 1).

She was admitted to the intensive care unit and started on an insulin drip with intravenous fluids. The echocardiogram revealed a reduced ejection fraction of 30%-35% with akinetic apex and hypokinetic septal segments suggesting Takotsubo cardiomyopathy (TC) (Video 1).

**Video 1:** Apical four-chamber view on echocardiogram showing reduced ejection fraction of 30%-35% with akinetic apex and hypokinetic septal segments suggestive of Takotsubo cardiomyopathy

Left heart catheterization showed mild nonobstructive coronary artery disease. The patient demonstrated marked improvement, with subsequent resolution of both acidosis and hypotension, leading to a transition to subcutaneous insulin therapy. Suspecting canagliflozin, which was started around one month prior to presentation, as the culprit for her euglycemic diabetic ketoacidosis (DKA), it was discontinued. Guideline-directed medical therapy (GDMT) with metoprolol succinate and losartan was initiated, and a follow-up echocardiogram in six weeks showed recovered ejection fraction with the complete resolution of wall motion abnormalities.

## Discussion

Sodium-glucose co-transporter-2 (SGLT2) inhibitors represent a significant advancement in the management of type 2 diabetes mellitus (T2DM). Approved by the US Food and Drug Administration (FDA) in March 2013, the first SGLT2 inhibitor, canagliflozin, revolutionized diabetes care by targeting renal glucose reabsorption at the proximal tubules, thereby enhancing urinary glucose excretion. This mechanism effectively lowers blood glucose levels by preventing hyperglycemia, offering a novel approach to glycemic control [4,5].

Despite their efficacy, SGLT2 inhibitors are not devoid of adverse effects. One major concern is the increased risk of ketoacidosis, including the paradoxical occurrence of euglycemic diabetic ketoacidosis (EDKA). While the overall incidence of DKA with SGLT2 inhibitors remains relatively low, large trials such as the DECLARE-TIMI 58 study and the CREDENCE trial have shown an increased probability of EDKA with SGLT2i as compared to placebo [6,7].

Several factors contribute to the heightened risk of DKA, including acute illness, starvation, and medication interactions. The mechanism involves the reduction of insulin secretion from pancreatic β-cells due to SGLT2 inhibition, leading to increased production of free fatty acids and subsequent ketone bodies through beta-oxidation in the liver. Furthermore, insulin typically inhibits the enzyme acetyl-coenzyme A (CoA) carboxylase, which generates malonyl-CoA, a potent inhibitor of carnitine palmitoyltransferase I (CPT-I). Since CPT-I facilitates the transport of fatty acids into mitochondria for beta-oxidation, reduced insulin levels stimulate CPT-I activity, further promoting ketone body production. Additionally, the administration of SGLT2 inhibitors may stimulate glucagon secretion, further promoting ketogenesis [5].

Though originally defined as DKA with a blood glucose level of <300 mg/dL, the concept of euglycemic DKA has evolved, now recognized at a blood glucose concentration of <200 mg/dL [4].

Dutta et al., in their systematic review and quantitative analysis comprising 47 articles, found that most reported patients diagnosed with EDKA were females (67.5%). Ethnicity was not consistently documented in most reports, though Asians were the most frequently mentioned (46.6%). Canagliflozin was the most commonly reported SGLT2 inhibitor (44%), followed by dapagliflozin (27%) and empagliflozin (25%). The analysis revealed a dose-dependent increase in cases of DKA associated with all three SGLT2 inhibitors. Additionally, concomitant medications frequently used alongside SGLT2 inhibitors included metformin, insulin, dipeptidyl peptidase 4 (DPP-4) inhibitors, and sulfonylureas, along with various vitamins and multivitamins [8].

Moreover, Dutta et al. [8] observed that patients taking canagliflozin were more prone to EDKA compared to those on dapagliflozin and empagliflozin. A multicentric cohort study by Ata et al. similarly identified canagliflozin as the most common SGLT2 inhibitor associated with EDKA [9]. This disparity may stem from canagliflozin being the earliest drug approved in its class, leading to its longer usage duration by physicians and consequently more extensive documentation of adverse events [8].

Furthermore, SGLT2 inhibitors have demonstrated significant cardiovascular and renal benefits, including reductions in heart failure (HF) hospitalizations and cardiovascular death [6]. In patients with heart failure, the clinical benefits of SGLT2 inhibitors were first established in those with reduced ejection fraction as the Empagliflozin Outcome Trial in Patients with Chronic Heart Failure and Reduced Ejection Fraction (EMPEROR-Reduced) and DAPA HF trials showed a reduction in the risk of HF hospitalization and death from cardiovascular causes [3]. The canagliflozin effect was specifically highlighted in the CANVAS trial where a lower risk of cardiovascular events was seen [3].

Moreover, recent advancements have positioned SGLT2 inhibitors as essential components of comprehensive heart failure management, with strong recommendations stemming from notable trials such as EMPEROR-Preserved and DELIVER. These trials showcased significant reductions in composite cardiovascular death or heart failure events, particularly in patients with heart failure exhibiting mildly reduced or preserved ejection fraction [10].

The paradoxical nature of adverse events with SGLT2 inhibitors is exemplified in our case report, where the side effect associated with the initiation of canagliflozin precipitated Takotsubo cardiomyopathy (TC), a stress-induced cardiomyopathy typically triggered by emotional or physical stressors. While SGLT2 inhibitors are recommended as a cornerstone in HF management, this case underscores the need for cautious prescribing, as the potential for adverse events, including TC, must be carefully weighed against the anticipated benefits [11].

## Conclusions

In summary, while SGLT2 inhibitors offer promising therapeutic avenues in diabetes and HF management, their use necessitates a nuanced approach. Clinicians must remain vigilant regarding potential adverse effects, including EDKA, particularly in high-risk populations. These findings emphasize the importance of personalized medicine and shared decision-making to optimize patient outcomes in the era of evolving pharmacotherapy.
